# Using systems medicine to identify a therapeutic agent with potential for repurposing in inflammatory bowel disease

**DOI:** 10.1242/dmm.044040

**Published:** 2020-11-27

**Authors:** Katie Lloyd, Stamatia Papoutsopoulou, Emily Smith, Philip Stegmaier, Francois Bergey, Lorna Morris, Madeleine Kittner, Hazel England, Dave Spiller, Mike H. R. White, Carrie A. Duckworth, Barry J. Campbell, Vladimir Poroikov, Vitor A. P. Martins dos Santos, Alexander Kel, Werner Muller, D. Mark Pritchard, Chris Probert, Michael D. Burkitt, The SysmedIBD Consortium

**Affiliations:** 1 Department of Cellular and Molecular Physiology, University of Liverpool, Liverpool L69 3GE, UK; 2 Faculty of Biology, Medicine and Health, University of Manchester, Manchester M13 9PL, UK; 3 geneXplain GmbH, Wolfenbuettel 38302, Germany; 4 LifeGlimmer GmbH, Berlin 12163, Germany; 5 Institute of Biomedical Chemistry, Moscow 119435, Russia; 6 www.sysmedIBD.eu

**Keywords:** Inflammatory bowel diseases, NF-κB, Drug repositioning, Interdisciplinary research, Organoids, Macrolide, Ulcerative colitis, Crohn's disease

## Abstract

Inflammatory bowel diseases (IBDs) cause significant morbidity and mortality. Aberrant NF-κB signalling is strongly associated with these conditions, and several established drugs influence the NF-κB signalling network to exert their effect. This study aimed to identify drugs that alter NF-κB signalling and could be repositioned for use in IBD. The SysmedIBD Consortium established a novel drug-repurposing pipeline based on a combination of *in silico* drug discovery and biological assays targeted at demonstrating an impact on NF-κB signalling, and a murine model of IBD. The drug discovery algorithm identified several drugs already established in IBD, including corticosteroids. The highest-ranked drug was the macrolide antibiotic clarithromycin, which has previously been reported to have anti-inflammatory effects in aseptic conditions. The effects of clarithromycin effects were validated in several experiments: it influenced NF-κB-mediated transcription in murine peritoneal macrophages and intestinal enteroids; it suppressed NF-κB protein shuttling in murine reporter enteroids; it suppressed NF-κB (p65) DNA binding in the small intestine of mice exposed to lipopolysaccharide; and it reduced the severity of dextran sulphate sodium-induced colitis in C57BL/6 mice. Clarithromycin also suppressed NF-κB (p65) nuclear translocation in human intestinal enteroids. These findings demonstrate that *in silico* drug repositioning algorithms can viably be allied to laboratory validation assays in the context of IBD, and that further clinical assessment of clarithromycin in the management of IBD is required.

This article has an associated First Person interview with the joint first authors of the paper.

## Abstract (precis)

**Summary:** Using state-of-the-art *in silico* drug discovery and real-time live-cell imaging techniques, we identify clarithromycin as a drug with potential for repositioning for inflammatory bowel disease.

## INTRODUCTION

Inflammatory bowel diseases (IBDs) affect 0.5-1.0% of people in the Western world and cause substantial morbidity and cost to society (Feagan et al., 2014; van Deen et al., 2014). The aetiology of IBD is complex. There are aberrant inflammatory responses, leading to mucosal damage and the disease phenotype.

Diverse therapeutic approaches are employed: many patients with mild ulcerative colitis are treated with mesalazine preparations, which predominantly act topically on the colonic mucosa to suppress inflammation (Marteau et al., 2005); others require systemic therapy with highly-specific biologic agents targeting inflammatory mediators (Baert et al., 1999; Targan et al., 1997). While these drugs have very different mechanisms of action, they influence host inflammatory responses and either directly or indirectly alter NF-κB signalling.

The NF-κB signalling network is a tightly controlled, dynamically regulated signal transduction pathway with several well-described transcription regulatory feedback loops (Lev Bar-Or et al., 2000); this orchestrates innate immune responses by regulating transcription through dimers of the five NF-κB proteins [RelA(p65), RelB, NF-κB1(p50), NF-κB2(p52) and c-Rel]. Signalling through this network is characterised by the oscillation of NF-κB proteins between the cytoplasm and nucleus, most clearly demonstrated for the RelA(p65) subunit (Nelson et al., 2004). In addition to changes in the inflammatory milieu, this network also affects several other processes that are dysregulated during chronic gastrointestinal inflammation, including cell turnover, DNA damage responses and cell senescence (Perkins, 2012). Several studies have associated aberrant NF-κB signalling with IBD (reviewed in Atreya et al., 2008; McDaniel et al., 2016; Merga et al., 2016).

Because of the complexity of NF-κB signalling, targeting specific components of the network has not been a successful drug development strategy to date, mainly because of the ubiquitous nature of NF-κB signalling. The impact of gross inhibition of critical members of the NF-κB signalling cascade has been unpredictable and associated with undesirable off-target effects. The complexity of NF-κB signalling during gastrointestinal tract inflammation is elegantly demonstrated by murine work. When transgenic mice lacking specific NF-κB subunits were subjected to dextran sulphate sodium (DSS) treatment in a model of colitis, mice lacking *cRel* and *Nfkb1* developed more severe colitis than wild-type controls, whereas *Nfkb2^−/−^* mice were resistant to colitis (Burkitt et al., 2015). C57BL/6 mice lacking IKKβ (also known as IKBKB) in epithelial cells were more severely affected by DSS colitis (Greten et al., 2004), whereas *Il-10*^−/−^ mice lacking IKKβ in the intestinal epithelium had similar colitis to those with intact NF-κB signalling; however, in both these models, loss of IKKβ in myeloid cells attenuated colitis and improved survival (Eckmann et al., 2008). It is therefore likely that the transcriptomic effects of attenuating NF-κB signalling in the epithelium and myeloid compartments are different.

This complexity has prompted calls for highly specific inhibitors that target components of the network in precisely selected cell types (Baud and Karin, 2009). This approach is useful in certain circumstances such as multiple myeloma, where targeting a specific cancer cell lineage is desirable and may be achievable. In contrast, for complex benign inflammatory diseases, including IBD, this strategy is less likely to be successful, owing to challenges in identifying a specific target cell population.

We propose an alternative strategy, to ‘nudge’ an individual's NF-κB signalling network towards an idealised healthy phenotype. This approach might offer a more pragmatic way of targeting aberrant NF-κB signalling, without the limitations of selective tissue targeting or gross pathway inhibition.

To investigate this in the context of IBD, we used a combination of novel bioinformatic analyses and laboratory studies to identify agents likely to impact NF-κB signalling and IBD. Because of the divergent roles of myeloid and epithelial NF-κB signalling during gastrointestinal tract inflammation, we established assays to measure the effects of drugs on both epithelial and immune tissues. We then validated the efficacy of the most highly ranked agent, both in terms of inhibiting NF-κB activity and modulating an *in vivo* model of colitis.

## RESULTS

**Fig. 1.**
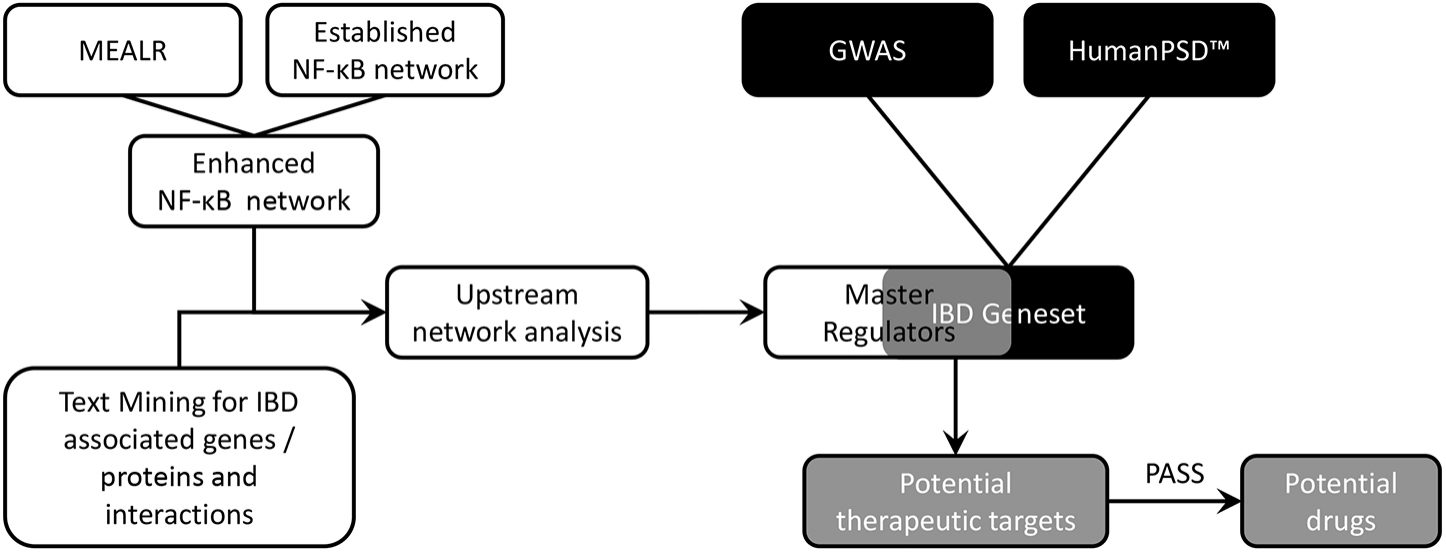
**Schematic representation of the bioinformatic approach** **used** **to identify drugs with the potential to modulate IBD and NF-κB signalling.** The black and white panels indicate the two different analysis streams, merging in the grey-shaded areas. GWAS, genome-wide association studies; IBD, inflammatory bowel disease; MEALR, motif enrichment analysis by logistic regression.

To predict drugs that can influence disrupted NF-κB signalling in IBD, a drug discovery strategy combining data from multiple sources [archived chromatin immunoprecipitation with sequencing (ChIP-seq) analyses, natural language text-mining of published abstracts, data from IBD genome-wide association studies (GWAS), and known IBD biomarkers and drug targets curated in the HumanPSD database] was developed (Fig. 1).

### Developing an enhanced NF-κB signalling network

Signalling molecules comprising (transcription) regulatory feedback loops present promising drug targets because they can influence the dynamics of a signalling pathway of interest, including the TNF/NF-κB pathway. To augment existing knowledge about NF-κB signalling, we developed an analysis workflow to identify genes encoding potential components of transcription regulatory feedback loops combining known NF-κB-involving signalling pathways, ChIP-seq assay-based NF-κB/RelA-bound genomic regions from the ENCODE project [Gene Expression Omnibus (GEO) series GSE31477], and a newly developed method to find combinations of enriched DNA sequence motifs [motif enrichment analysis by logistic regression (MEALR)] (see Materials and Methods for details). Combinations of prioritised motifs tend to coincide with transcription factors that are known to cooperate. Our analysis found 24 transcription factors that appear to cooperate in NF-κB signalling within the genomic regions reported by ENCODE (Table 1) as well as 90 potential NF-κB/RelA-target genes that encode components of known pathways (Table 2). The results were used to annotate the TRANSPATH^®^ database of mammalian signal transduction and metabolic pathways.

**Table 1.**
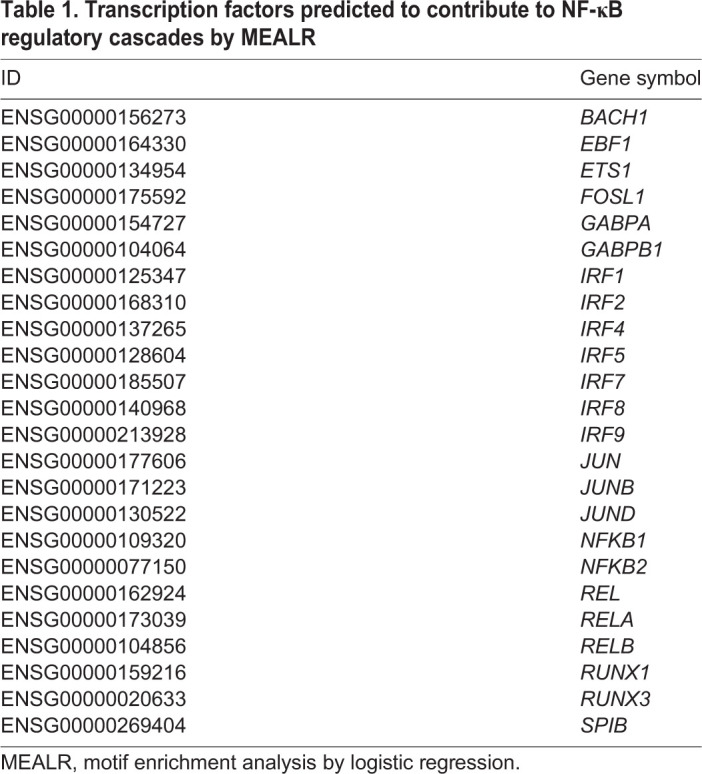
**Transcription factors predicted to contribute to NF-κB regulatory cascades by MEALR**

**Table 2.**
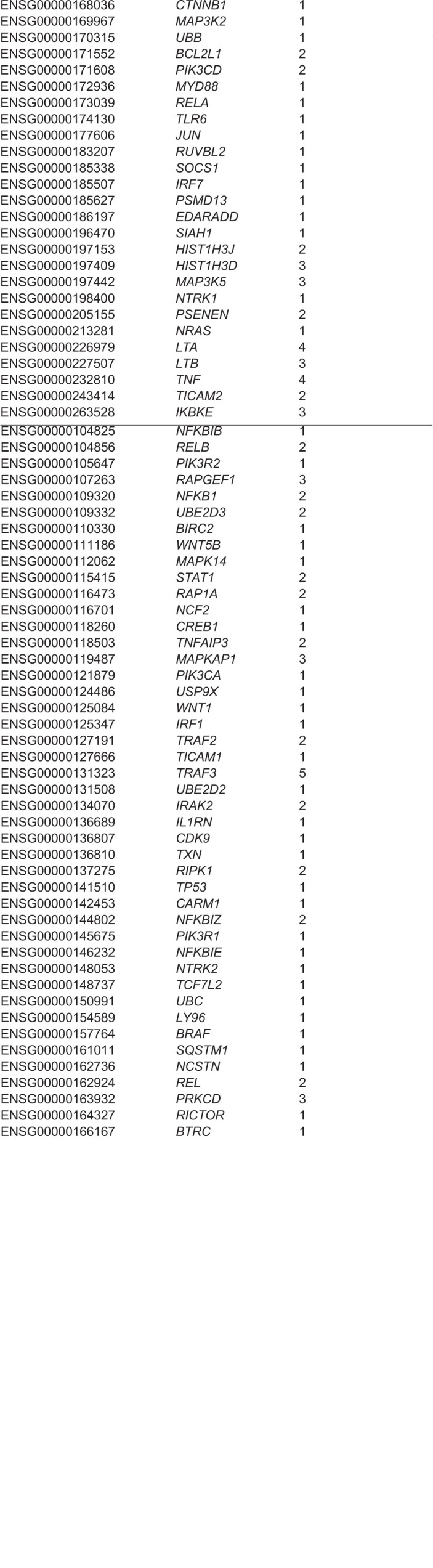
**NF-κB target genes identified by MEALR to be involved in regulatory cascades**

### Text mining to establish context proteins and genes for upstream network analyses

One thousand relevant abstracts were retrieved using the MedlineRanker tool (Gijón-Correas et al., 2014) from the PubMed database using the Medical Subject Headings (MeSH) terms ‘Inflammatory bowel diseases’ and ‘NF-κB’. Protein–protein interactions were identified in these abstracts using PESCADOR (Barbosa-Silva et al., 2011). PESCADOR detects genes, proteins and their interactions, and rates them based on co-occurrences in an abstract. Using this tool, 827 interactions common for both *Homo sapiens* and *Mus musculus*, two for *Mus musculus* alone and 26 uniquely for *Homo sapiens* were extracted (Table S1). After querying the TRANSPATH^®^ database for known direct interactions, 127 novel co-occurrences of genes or proteins in the abstracts were analysed. This table of interactions was used to provide context to subsequent upstream network analysis to impute the key regulatory nodes for NF-κB signalling in IBD.

### Identifying master regulators of the NF-κB signalling network

An upstream search of the various molecular components of NF-κB complex – including NF-κB1-isoform1, NF-κB1-isoform2, NF-κB2-isoform4, NF-κB2-p100, NF-κB2-p49, RelA-p35, RelA-p65-delta, RelA-p65-delta2, RelA-p65-isoform1, RelA-p65-isoform4, RelB and c-Rel – was performed. The network search extended to a maximal radius of ten steps upstream of the NF-κB components and used a false discovery rate (FDR) cut-off of 0.05 and *Z*-score (reflecting how specific each master regulator was) cut-off of 1.0. The list of interacting proteins obtained by text mining was used to provide ‘Context proteins’ for the master-regulator acquisition algorithm (Kel et al., 2016). This analysis revealed 325 controlling nodes predicted to exert signalling activity for the NF-κB components (Table S2).

### Developing a list of IBD-associated genes and potential therapeutic targets

A list of IBD-associated genes and potential therapeutic targets was established by retrieving information from the HumanPSD database, including known IBD biomarkers and drug targets, and two lists of IBD-related genes from GWAS, one focused on revealing genes of IBD prognosis (Lee et al., 2017) and another on IBD susceptibility (Hasler et al., 2017). This identified 159 IBD targets, with an indication of the source of evidence about their relevance to IBD (Table S3).

Finally, to produce a list of proposed therapeutic targets, the list of IBD targets was intersected with the 325 controlling nodes to obtain 62 candidates (defined as IBD key nodes; Table S4) that represent genes predicted to influence IBD-associated NF-κB regulation, which had also independently been identified as IBD-associated genes or potential therapeutic targets.

### Predicting drugs that influence the IBD key nodes

The PASS software package was used to predict drugs that interact with IBD key nodes. This software predicts the ability of a chemical structure to interact and influence the activity of defined molecular targets and biological activities. The 62 key nodes were translated into 46 PASS activities (Tables S5 and S6). To identify compounds with potential for clinical repositioning, the top 200 drugs library was analysed to predict the probability that these established and licensed agents interfere with each PASS activity.

A cumulative score was calculated across all 46 PASS activities for each drug. We also required that the drugs were predicted to be active against the PASS activity ‘Transcription factor NF-κB inhibitor’, and 29 compounds achieved these criteria (Table 3). Importantly, this table included several corticosteroids already used to treat IBD, supporting the validity of the discovery strategy.

**Table 3.**
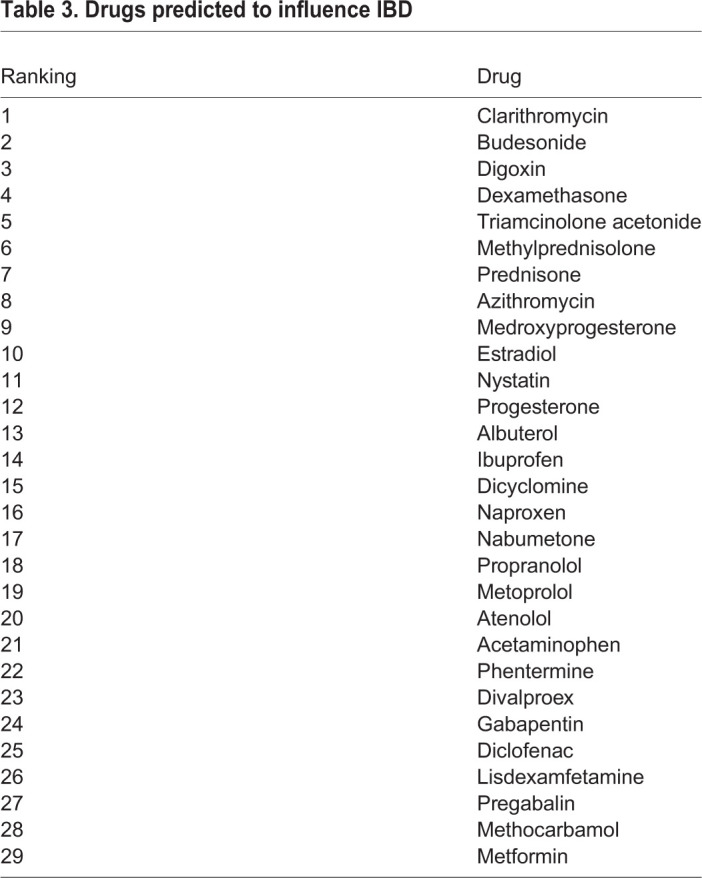
**Drugs predicted to influence IBD**

The highest-ranked drug was a macrolide antibiotic, clarithromycin (CLA). This agent is of particular interest because macrolides have an established role in treating aseptic inflammatory conditions, including chronic rhinosinusitis (Oakley et al., 2017) and panbronchiolitis (Lin et al., 2015), and because CLA has previously been trialled in IBD with divergent outcomes (Leiper et al., 2008, 2000). We, therefore, considered it to be an excellent candidate for strategic repurposing and have used CLA as a paradigm molecule for the development of a mechanism-led drug validation pathway.

### CLA suppresses stimulus-induced luciferase activity in primary cell cultures

**Fig. 2.**
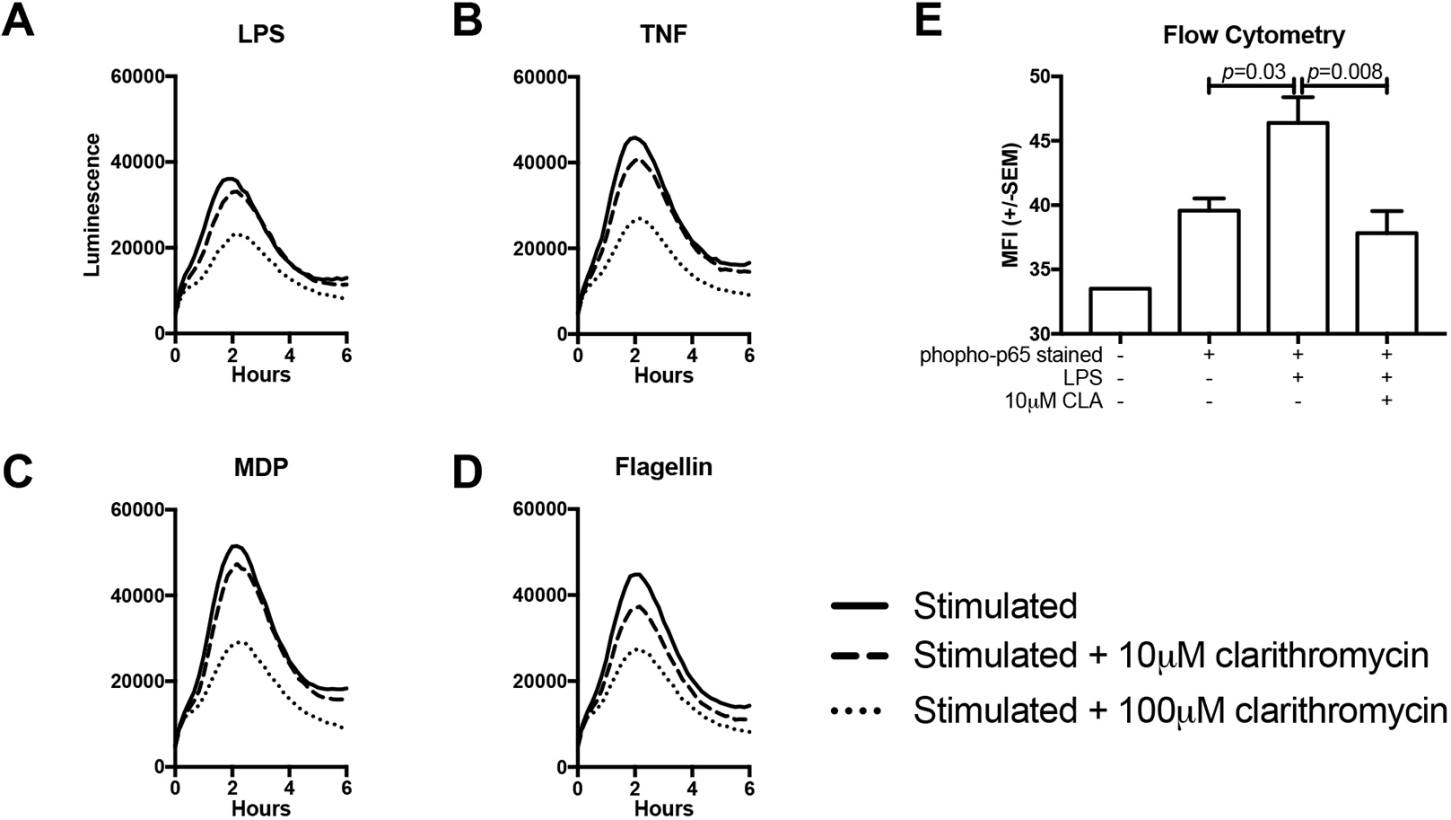
**CLA suppresses stimulus-induced NF-κB activation in primary murine cell cultures.** (A-D) Luciferase activation curves for peritoneal macrophages extracted from HTNF.LucBAC mice stimulated with 10 ng/ml lipopolysaccharide (LPS) (A), 40 ng/ml TNF (B), 10 µg/ml muramyl dipeptide (MDP) (C) or 500 ng/ml flagellin (D). Solid lines indicate the responses for cells pre-treated with drug vehicle (DMSO); dashed and dotted lines indicate the responses generated from cells pre-treated with 10 µM or 100 µM clarithromycin (CLA), respectively. (E) Median fluorescence intensity for anti-phopsho-p65 antibody-stained peritoneal macrophages from C57BL/6 mice either unstimulated or stimulated with LPS and pre-treated with 10 µM CLA or DMSO vehicle. *N*=4 mice. Statistically significant differences tested by one-way ANOVA and Dunnett's post hoc test.

To validate the efficacy of CLA as an inhibitor of NF-κB-mediated transcription, primary cultures from the hTNF.LucBAC mouse, a transgenic line that expresses firefly luciferase under the control of the entire human *TNF* promoter (Minshawi et al., 2019), were used. Luciferase activity was triggered by ligand binding to the TNF receptor and pattern recognition receptors including TLR4 [lipopolysaccharide (LPS)], TLR5 (flagellin) and NOD2 [muramyl dipeptide (MDP)] (Fig. 2A-D) in peritoneal macrophages harvested from this mouse. When cells were pre-treated for 30 min with 10 µM or 100 µM CLA, a dose-dependent reduction in luciferase activity was observed, independent of the stimulus applied.

The effect of CLA on NF-κB activation in murine peritoneal macrophages was confirmed by isolating peritoneal macrophages from four wild-type (WT) mice, and treating them with 10 µM CLA or vehicle before stimulation with 1 µg/ml LPS. Cells were fixed, stained for phosphorylated p65 and quantified by flow cytometry. Median fluorescence intensity (MFI) increased on stimulation (*P*=0.03, two-way ANOVA); co-administration of LPS and CLA suppressed this response (*P*=0.008; Fig. 2E).

**Fig. 3.**
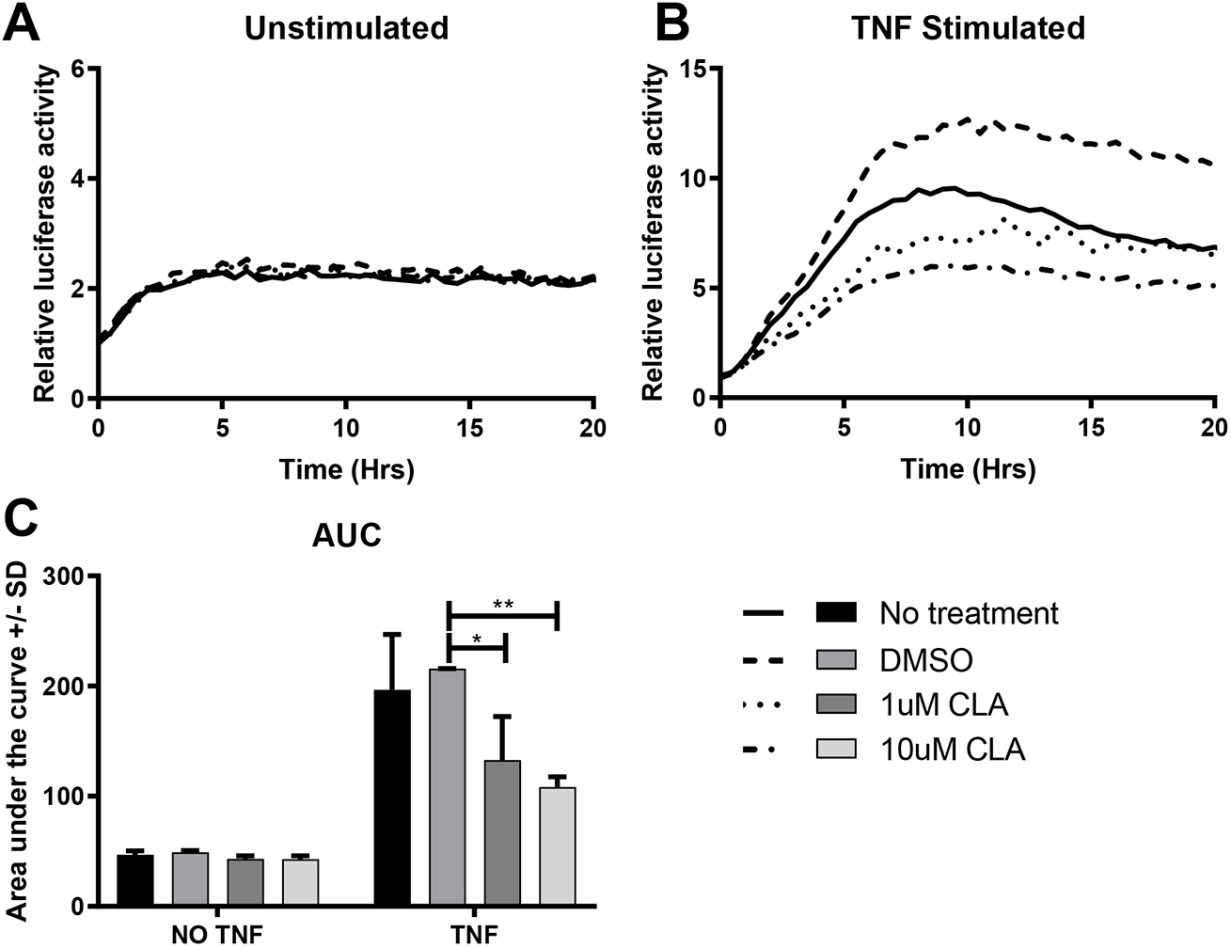
**CLA suppresses TNF-induced luciferase activity in enteroids.** (A,B) Representative luciferase activation curves for enteroids derived from HTNF.LucBAC mice either unstimulated (A) or stimulated with 100 ng/ml TNF (B), and without pre-treatment (solid line), or with 30 min pre-treatment with DMSO vehicle (dashed line), 1 µM CLA (dotted line) or 10 µM CLA (dotted and dashed line). (C) Area under the curve (AUC) calculations for the same experiment. *N*=3. *P<0.05, **P<0.01. Statistically significant differences tested by two-way ANOVA and Dunnett's post hoc test.

Small intestinal organoids (enteroids) were established from hTNF.LucBAC mice to determine whether CLA could also alter NF-κB responses in gastrointestinal epithelial cell cultures. Luciferase activity was detectable in these cultures in response to 100 ng/ml TNF administration (Fig. 3A,B). Luciferase activity was significantly suppressed by pre-treatment for 30 min with either 1 µM (*P*=0.014) or 10 µM (*P*=0.001) CLA (Fig. 3B,C).

### CLA suppresses TNF-induced NF-κB (p65) shuttling in enteroids

To assess whether CLA influenced NF-κB protein shuttling dynamics, enteroid cultures from reporter mice expressing p65-DsRedxp/IκBα (also known as NFKBIA)-eGFP were established. This mouse expresses human p65-DsRedxp and human IκBα-eGFP fusion proteins. We used these organoids in live-cell confocal imaging studies to observe the nuclear translocation of p65 in real time. Images were analysed using CellTracker software (Ashall et al., 2009), which allows painstaking quantification of nuclear shuttling of fluorescently labelled proteins.

**Fig. 4.**
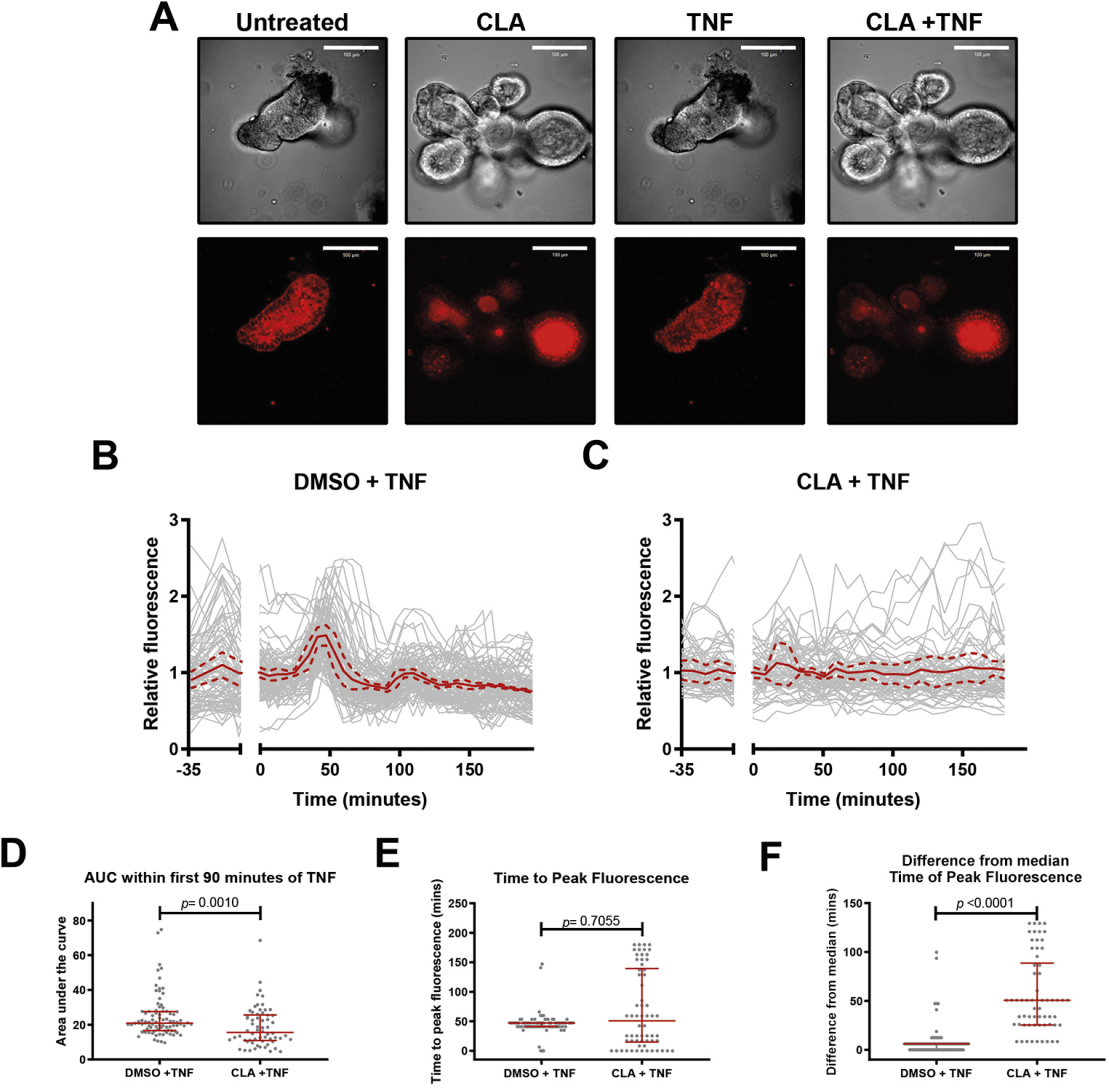
**CLA suppresses TNF-induced p65 nuclear translocation in enteroids.** (A) Representative images of brightfield (top row) and red channel (bottom row) images of dynamic, live-cell imaging studies of enteroids derived from p65-DsRedxp/ IκBα-eGFP mice, either untreated, treated with CLA alone, treated with 100 ng/ml TNF alone, or pre-treated with 10 µM CLA and subsequently stimulated with TNF. (B,C) Relative nuclear red fluorescence curves for individual cells (grey lines), mean (solid red lines) and 1 s.d. above and below the mean (dashed red lines) over time. Cells were pre-treated with DMSO vehicle (B) or CLA (C) at time −35 min and stimulated with TNF at time 0 min. (D) AUC calculations for individual cells between time 0 min and time 90 min for B and C. Statistically significant differences tested by Mann–Whitney *U-*test. (E) Time to peak fluorescence, post-TNF stimulation for individual cells; statistically significant differences tested by Mann–Whitney *U-*test. (F) Difference of individual cells’ peak fluorescence from the median value; statistically significant differences tested by Mann–Whitney *U-*test. *N*=88 vehicle pre-treated cells, *N*=62 CLA pre-treated cells for D-F; lines represent median and IQR for these panels. Scale bars: 100 µm.

Administration of 100 ng/ml TNF induced synchronised p65 translocation to the nucleus of cells within enteroids with a periodicity of ∼50 min. This was observed as a highly damped shuttling response, with a single wave of synchronised nuclear translocation, followed by a second wave of partially synchronised translocation, after which further nuclear localisation occurred in an apparently stochastic fashion (Fig. 4A,B; Movie 1). When cultures were pre-treated with 10 µM CLA for 30 min, p65-DsRedxp nuclear translocation was markedly suppressed (Fig. 4C).

To quantify these events more precisely, the mean area under the curve (AUC) during the first oscillatory wave (Fig. 4D) was calculated, and demonstrated decreased nuclear intensity of p65-DsRed fluorescence in CLA pre-treated enteroids (*P*=0.005). The time to peak nuclear fluorescence after 100 ng/ml TNF administration was also quantified (Fig. 4E). In dimethyl sulfoxide (DMSO) vehicle-treated cells, peak fluorescence occurred at a median time of 47.2 min [interquartile range (IQR) 41.0-47.2 min]. This was not significantly different for CLA-treated enteroids, but there was significantly greater difference from the median time of peak fluorescence observed in CLA-treated than in vehicle-treated cells (*P*<0.0001; Fig. 4F). To assess synchronisation of nuclear translocation, the time between peak fluorescence and median time for peak nuclear fluorescence was calculated for each cell (Fig. 4F). This confirmed an 8-fold greater variation in timing for peak nuclear fluorescence in CLA-treated enteroids compared to controls (*P<*0.0001), confirming that the synchronisation of p65 nuclear translocation was lost following exposure to CLA.

### CLA suppresses LPS-induced NF-κB (p65) DNA binding *in vivo*

To determine whether the effects of CLA on stimulus-induced NF-κB activity observed *in vitro* also occurred *in vivo*, 0.125 mg/kg LPS was administered to groups of six C57BL/6 male mice by intraperitoneal injection, either with or without CLA co-administration. This stimulus induces small intestinal epithelial cell shedding, regulated by both NF-κB1 and NF-κB2 signalling pathways (Williams et al., 2013).

**Fig. 5.**
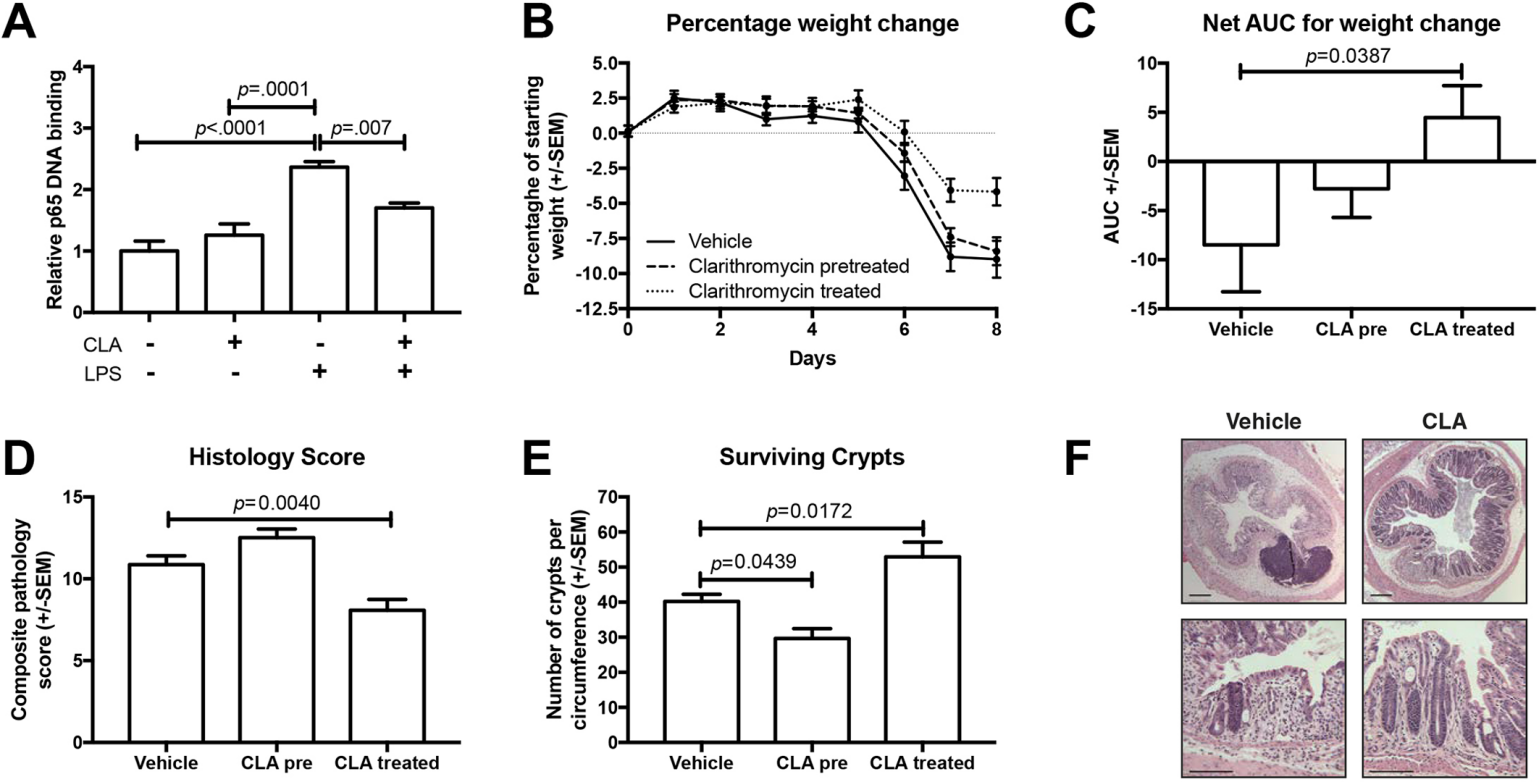
**CLA attenuates NF-κB activation and dextran sulphate sodium (DSS) colitis in C57BL/6 mice.** (A) Relative p65 DNA binding activity in whole-cell lysates from the proximal small intestine of C57BL/6 mice pre-treated for 3 days with 50 mg/kg CLA or saline vehicle, and subsequently injected i.p. with 0.125 mg/kg LPS or vehicle. *N*=5-6. (B-F) Effect of CLA on outcomes of DSS colitis in C57BL/6 mice. (B) Weight loss plotted over time. (C) AUC analysis of weight loss. (D) Histology severity scores. (E) Number of surviving crypts per colonic circumference. (F) Representative photomicrographs of the colonic mucosa of DSS-treated mice co-treated with saline vehicle or 10 mg/kg CLA by oro-gastric gavage. *N*=9-10. Statistically significant differences tested by one-way ANOVA and Dunnett's post hoc test in all panels. Scale bars: 200 µm (top row in F) and 100 µm (bottom row in F).

LPS administration induced a 2-fold increase in p65 DNA binding, compared to saline vehicle control (*P*<0.001, one-way ANOVA and Dunnett's post hoc test); pre-treatment with CLA suppressed this effect by ∼51% (*P*=0.007; Fig. 5A).

### CLA suppresses DSS-induced colitis

To determine whether CLA affected murine colitis *in vivo*, CLA or vehicle was administered to mice receiving DSS to induce colitis. Animals received CLA or vehicle daily for 4 days by oro-gastric gavage; after a 4-day washout period, 2.5% w/v DSS in drinking water *ad libitum* was commenced for 5 days, followed by recovery for a further 3 days.

Mice co-administered DSS and CLA lost significantly less weight than other groups (*P*=0.039, one-way ANOVA and Dunnett's post hoc test; Fig. 5B,C) and had lower compound histology scores (*P*=0.004; Fig. 5D,F) and a higher number of surviving colonic crypts than mice treated with vehicle (*P*=0.017; Fig. 5E). These results suggest that CLA at least partially ameliorates this model of colitis.

### CLA suppresses TNF-induced NF-κB (p65) nuclear localisation in human enteroids

**Fig. 6.**
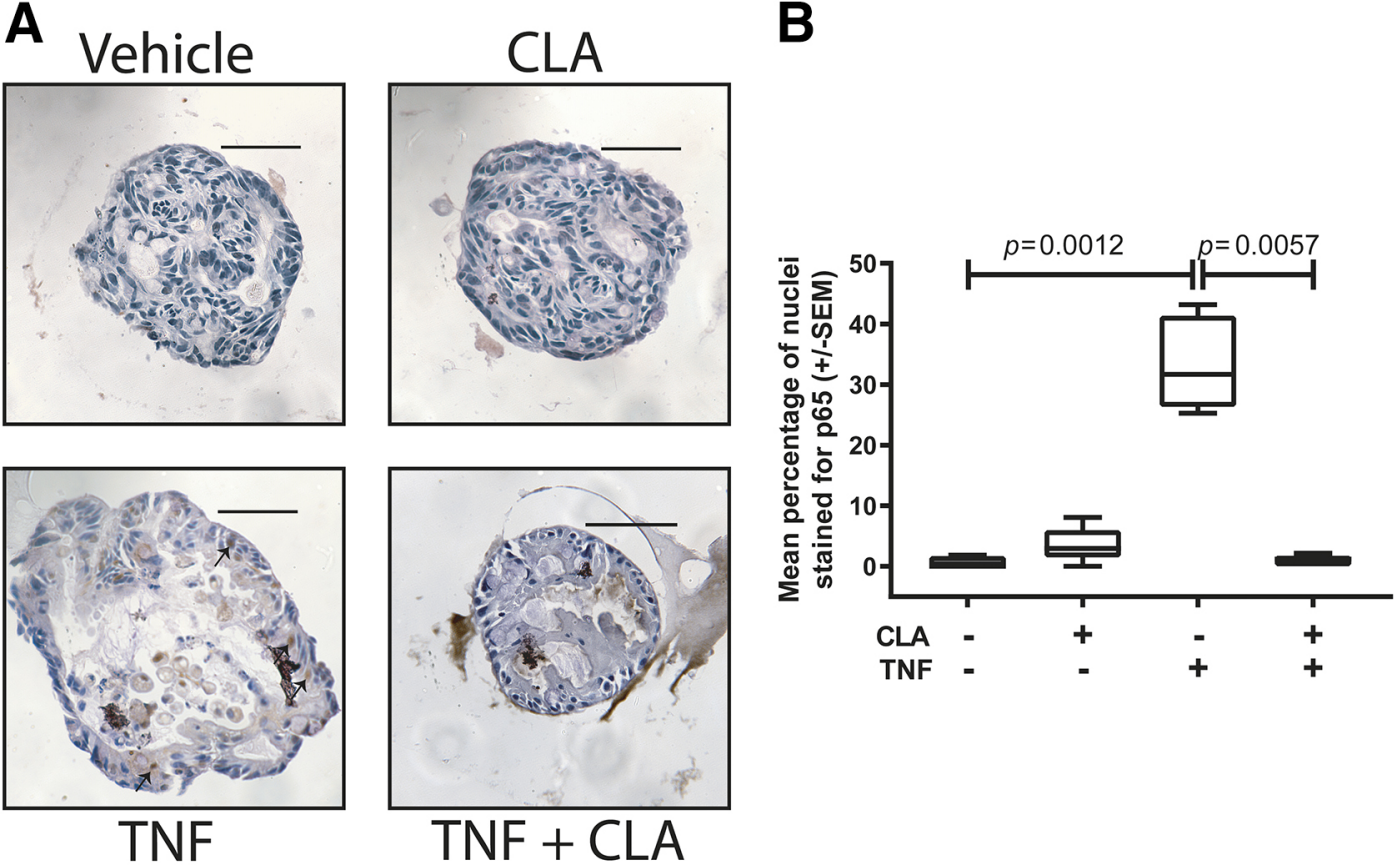
**CLA attenuates NF-κB activation in human enteric organoids.** (A) Representative photomicrographs of human ileal enteroids either unstimulated or stimulated with 100 ng/ml TNF, and either co-administered DMSO vehicle or 10 µM CLA and immunostained for total p65. (B) Quantification of nuclear p65 staining of human enteroids. Statistically significant differences tested by Kruskal–Wallis one-way ANOVA and Dunn's post hoc. *N*=6. Scale bars: 50 µm.

To determine whether the effects identified in murine primary culture and *in vivo* experiments were also relevant to humans, passaged human ileal organoids from individuals with no evidence of IBD were pre-treated with 10 µM CLA or DMSO vehicle for 30 min before stimulation with 100 ng/ml TNF. Paraformaldehyde-fixed cultures were immunostained for p65, and the percentage of cells expressing nuclear-localised p65 was quantified. In untreated human enteroids, 0.6±0.37% (±s.e.m.) of cells demonstrated p65 nuclear localisation, administration of TNF induced a 57-fold increase in cells expressing nuclear p65 (33±3.2%, *P*<0.0001, one-way ANOVA and Dunnett's post hoc test, *N*=6 per group). Pre-treatment with CLA suppressed TNF-induced nuclear localisation of p65 to a level comparable with DMSO vehicle-treated enteroids (1±0.36%, *P*<0.0001; Fig. 6).

## DISCUSSION

Our study results indicate that the macrolide antibiotic CLA is a modifier of NF-κB signalling in the gastrointestinal tract. Identification of CLA, using an *in silico* screen of licensed drugs, demonstrates how novel bioinformatics can be used to progress drug repurposing, a strategy that has the potential to reduce the cost of future drug development.

The SysmedIBD Consortium-integrated diverse skill sets to develop this approach could be applied in different ways in the future. An identical bioinformatic analysis could be used to screen panels of small molecules to identify novel therapeutics for IBD. Similarly, the system could be adapted for use in other contexts in which NF-κB signalling is of paramount importance or refocussed onto different signalling networks.

Our list of drugs predicted to influence IBD outcomes included several drugs in routine use for IBD, most prominently the corticosteroids, which are used to treat acute relapses of IBD (Truelove and Witts, 1955; Turner et al., 2007). The analysis also identified sex hormones including medroxyprogesterone and oestradiol, which have been shown to modulate colitis (Armstrong et al., 2017), and colitis-associated adenoma development *in vivo* (Son et al., 2019), as well as non-steroidal anti-inflammatory drugs (NSAIDs), which, in clinical practice, are identified as agents that exacerbate IBD (Long et al., 2016). The harmful effects of NSAIDs result from the inhibition of constitutively expressed COX-1 in the gastrointestinal epithelium, causing epithelial damage and ulceration; inhibition of COX-2, which is upregulated at sites of inflammation is a well-established anti-inflammatory mechanism that influences NF-κB signalling. The drug discovery approach deliberately included a bias for agents that alter NF-κB signalling; it is likely that NSAIDs have been selected because of this bias.

These observations demonstrate the importance of interdisciplinary working. The *in silico* drug discovery model is a powerful tool to identify drugs that may be repurposed, but decisions about which agents to pursue for further analysis can only be made in the context of existing clinical literature.

Laboratory evaluation of CLA aimed to demonstrate proof of principle that a drug identified by ­*in silico* testing would demonstrate the predicted mechanism of action and show efficacy *in vivo*. Our strategy has potential for development into a higher-throughput compound screening system. For example, the hTNF.LucBAC macrophage assay can be performed in a 96-well plate format, and peritoneal macrophages are abundant, simple to extract and highly sensitive to stimulus. Reporter enteroids generated from hTNF.LucBAC mice allowed us to validate the findings seen in peritoneal macrophages in a relevant, untransformed epithelial model using comparable technology, but are unlikely to be amenable to high-throughput assay development, owing to the challenges (and cost) of maintaining a 3D culture in a small well in a culture plate.

The visualisation of p65.DsRed translocation between the nucleus and cytoplasm in enteric organoids is technically challenging, and not immediately scalable. One of the challenges that we persistently encountered was fluctuating fluorescence prior to stimulation with TNF, despite efforts to rest the cells prior to imaging. Unlike a monoculture of immune cells, enteroids are a complex system with several cell types represented within the organoid structure. Whether this is due to paracrine secretion between different cells within the organoid structure or is a factor related to organoid culture conditions is not possible to dissect currently. Our assays also demonstrated fundamental differences in NF-κB signalling dynamics compared to cancer cell lines (Harper et al., 2018; Nelson et al., 2004), with TNF-induced p65 oscillations being heavily damped in organoids. This observation demonstrates the value of untransformed primary culture; the mechanisms underlying these differences will be subject to further investigation.

The observation that intestinal NF-κB signalling is altered by CLA *in vivo* is in keeping with earlier work using cancer cell lines (Peng et al., 2014), but it is the first demonstration that macrolides alter this signalling pathway in either untransformed enteroids or gastrointestinal mucosae *in vivo*. Previous studies demonstrated that a non-antibiotic macrolide, CSY0073, influenced acute DSS colitis in C57BL/6 mice (Mencarelli et al., 2011), but CLA had not been studied. Our murine experiments were complicated by the antibiotic effects of CLA. Murine models of colitis are known to vary depending on animal housing conditions and host enteric microbiota. Several strategies could have been adopted to help differentiate the antibiotic and anti-inflammatory effects of CLA, all of which are flawed. Germ-free mice and gnotobiotic mice have been used to impute impacts of gut microbiota in IBD models, but they are flawed as immune systems development is divergent in germ-free mice. Broad-spectrum antibiotics have been used in an attempt to eradicate commensal enteric bacteris, but no antibiotic combination will effectively achieve this goal, and its effect on the mycobiome and virome would be unquantifiable.

We preferred to adopt a consistent approach that was based on pre-exposure to the CLA. The intention of this approach was to build evidence for the antibiotic effect of CLA by examining the impact of pre-treatment with CLA, and comparing it to the anti-inflammatory effects of sustained CLA treatment. In the LPS administration experiment, the time points are extremely short; therefore, an antibiotic effect within the experimental period is highly unlikely to explain differences between CLA-pre-treated animals and animals administered CLA immediately before LPS. The antibiotic effect is a greater concern in the DSS experiment, but the inclusion of data from relevant control animals has allowed us to unpick the anti-inflammatory effect. Intriguingly the histological damage in mice pre-treated with CLA was worse than that seen in the vehicle control mice, suggesting that the antibiotic effect in this model might, if anything, have the opposite effect to sustained CLA treatment.

The *in vivo* studies rely on studying the whole organism, and it was not feasible to separate epithelial and immune compartments during this study; this was one of the prime motivations for studying immune and epithelial models *in vitro*. Our final validation was to characterise whether CLA influenced human epithelial NF-κB signalling. We investigated this using a HeLa reporter cell line model, which is fast, inexpensive, commercially available and could be adapted as a high-throughput screening test (Fig. S2), but it is inferior to the hTNF.LucBAC mouse primary culture models as NF-κB signalling is dysregulated in many cancer cell lines. By generating human enteroids, the effects of CLA on a primary, untransformed human epithelial cell culture could be assessed. Unfortunately, the assay used for NF-κB activation in this model was necessarily less specific, but the results supported those obtained with other assays.

It was serendipitous that the highest-ranked agent identified for repurposing was a drug that had already been trialled in IBD. Importantly the outcomes of previous trials of CLA in IBD have been heterogeneous (Goodgame et al., 2001; Graham et al., 1995; Leiper et al., 2008, 2000; Selby et al., 2007), suggesting that there may be context-dependent factors that determine whether CLA is useful in a group of patients.

Four published papers and a conference abstract (Graham et al., 1995) have reported the effect of CLA in IBD: they all focussed on Crohn's disease and were predicated on an antibiotic effect of CLA, either targeting intra-macrophage killing of *Escherichia* *coli* (Leiper et al., 2008, 2000) or attempting to eradicate *Mycobacterium avium paratuberculosis* (Goodgame et al., 2001; Graham et al., 1995; Selby et al., 2007). Several factors may explain the discordant outcomes: in all studies on the effects of CLA on IBD, patient inclusion was based on clinical definitions of active IBD and response assessed by clinical outcome measures. These measures lack objectivity and would not be acceptable endpoints or selection criteria for a current study.

Selby et al. (2007) and Goodgame et al. (2001) assessed the long-term effect of bacterial eradication; any anti-inflammatory effect would have been lost during the prolonged follow-up period before the primary endpoint was assessed. Leiper et al. (2000, 2008) examined an earlier time point when anti-inflammatory effects could have been observed, but were not the focus of the study. Two separate cohorts of patients with clinically active Crohn's disease were recruited from a single centre; in the first study, CLA appeared to improve patient outcomes, but the same was not observed during the second study. At the time, the divergent outcomes were explained by the small size of the initial study and the relatively soft criteria for inclusion of patients with active IBD. These explanations could still be valid, but an alternative hypothesis is that a subgroup of patients with active disease might respond to CLA. One of the motivations for investigating existing drugs during this study was specifically to identify agents that have equivocal evidence based on traditional trials but that have evidence for an untested mechanism of action.

The current study confirms that, in addition to its antibiotic effect, CLA has anti-inflammatory properties, which are relevant to gastrointestinal epithelia. Further carefully designed clinical studies will be needed to test the anti-inflammatory effects of treatment with CLA. Earlier discordant trial results raised questions about trial design and patient selection. In the current era of precision medicine, it is important that patients are carefully selected for treatments, and that agents with previous dichotomous clinical trial results are revisited. To effectively review drugs for precision use, their mechanisms of action need to be understood. This study has helped us to understand how CLA affects NF-κB signalling dynamics, and we hypothesise that it should be possible to select a group of CLA-responsive patients based on their NF-κB signalling status.

Our Consortium has recently shown that patients with IBD cluster into several different cohorts based on a dynamic measure of NF-κB responses in peripheral blood monocyte-derived macrophages (Papoutsopoulou et al., 2019). We hypothesise that, by using this new measure of NF-κB activity, it will be possible to identify IBD patients most likely to respond to NF-κB-targeted therapy in the form of CLA, and thereby leverage a precision medicine trial.

In conclusion, our findings strongly suggest that CLA could be a viable, anti-inflammatory therapeutic agent for IBD. In order to progress to a personalised medicine trial of CLA in IBD, a partner diagnostic test that can demonstrate altered NF-κB dynamics in patients' peripheral blood monocyte-derived macrophages is under development (Papoutsopoulou et al., 2019). Once established, this will be used to inform a personalised drug repurposing trial for CLA in IBD.

## MATERIALS AND METHODS

### MEALR

Transcription regulatory feedback loops driven by NF-κB/RelA and cooperating transcription factors were inferred based on existing knowledge about signalling pathways involving NF-κB/RelA transcription factor-binding motifs and ChIP-seq experiment data targeting NF-κB/RelA-bound sites in the human genome. The analyses were mainly conducted using the geneXplain platform (Koschmann et al., 2015).

Our analysis aimed to identify feedback loops that encompass components of NF-κB/RelA pathways as well as cooperating transcriptional regulators that are themselves regulated by NF-κB/RelA (Fig. S1). First, we applied a novel method for MEALR. MEALR was developed to analyse binding site combinations in sets of DNA sequences that are likely to be bound by a common set of transcriptional regulators, as obtained through ChIP-seq experiments. The algorithm chooses a small set of DNA sequence motifs from a possibly large library of such models. Given *N* sequences assigned to classes *y*_i_∈{0, 1} and a library of *M* positional weight matrices (PWMs), MEALR estimates a sparse logistic regression (LR) model:
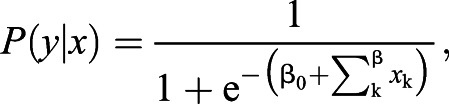
using the vector of sequence scores calculated for the ith sequence:
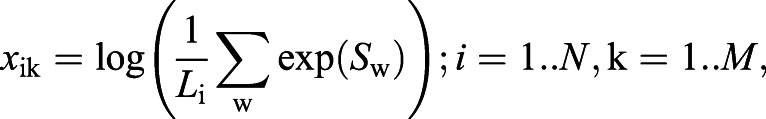


where *S*_w_ is the log-odds score of the PWM assigned to the wth sequence window. The model coefficients can be used to prioritise motifs of transcriptional regulators with respect to their importance for experimentally observed binding events. Combinations of motifs prioritised by MEALR tend to coincide with transcription factors that are known to cooperate.

We applied MEALR to reveal discriminative DNA sequence motifs in genomic regions bound by NF-κB/RelA after TNF stimulus. Sequence scores were calculated for a subset from the TRANSFAC(R) database, release 2014.4, consisting of 1429 motifs for transcription factors from vertebrate organisms. Genomic NF-κB/RelA binding sites were identified by analysis of ChIP-seq experiments carried out by the ENCODE project and deposited in GEO [Gm10847, Gm12878, Gm12891, Gm12892, Gm15510, Gm18505, Gm18526, Gm18951, Gm19099, and Gm19193 (GEO series GSE31477)]. MEALR models were estimated to distinguish ChIP-seq peak sequences from genomic background sequences that were randomly sampled from gene promoter regions not overlapping with peaks of the respective experiment. The analysis was conducted five times for each ChIP-seq data set with different background sequence sets. We retained motifs that were incorporated in the model in all five runs. As MEALR may select similar binding motifs with different identifiers in separate runs, we considered DNA-sequence motif similarity quantified by our method m2match (Stegmaier et al., 2013).

A total of 62 TRANSFAC(R) motifs were selected by MEALR in at least nine of the ten ChIP-seq data sets. These motifs mapped to 38 transcription factor genes including NF-κB-type factors as well as other transcription factors that our analysis suggests cooperate with NF-κB (Table S1).

To identify potential consensus target genes of NF-κB/RelA, we grouped overlapping peak regions and mapped groups with peaks in at least six out of ten ChIP-seq experiments to nearby genes. This part of the analysis focused on peaks with a maximal length of 3000 bases (>99.1% of all peak regions in the ten ChIP-seq experiments). We identified 4835 consensus ChIP-seq regions in the vicinity of 6329 genes. These genes were defined as consensus target genes.

Among the target genes, we identified 24 transcription factors inferred by MEALR, as well as 90 of 284 genes encoding components of signalling pathways and cascades involving NF-κB/RelA collected from the TRANSPATH(R) database, release 2013.3. These comprise the feedback loops with NF-κB/RelA consensus targets described in Fig. S1.

### Pathway analysis for master regulator search

Molecules that regulate the expression of differentially expressed genes through control of the activity of NF-κB subunits were defined as master regulators and were identified by applying a key node analysis algorithm (Kel et al., 2006), using the TRANSPATH^®^ database of gene regulatory and signal transduction pathways (Krull et al., 2006). Key nodes were prioritised based on the weighted ratio between the number of molecules from the input set that could be reached from the key node in up to ten steps and the total number of reachable nodes. The higher the score, the greater the chance that the key node plays a master regulatory role.

### *In silico* drug discovery

Relationships between the chemical structure of compounds and their biological activities were analysed using large-scale prediction of activity spectra (Filimonov et al., 1995; Poroikov et al., 2001) (http://genexplain.com/pass/) to discover potential new drugs for IBD treatment. The PASS algorithm is based on Bayesian estimates of probabilities of molecules belonging to the classes of active and inactive compounds (Filimonov et al., 2014). The predicted activity spectrum is presented in PASS by the list of activities with probabilities ‘to be active’ *P*_a_ and ‘to be inactive’ *P*_i_ calculated for each activity.

For each compound of the tested library, a cumulative score was computed across the 46 PASS activities selected in the previous analysis:
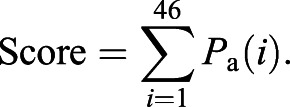


Here *P*_a_(*i*) is the probability for the given compound to be active for each activity *i*. For each compound tested, the cumulative score was required to be higher than 3.0, and the *P*_a_ for the PASS activity ‘Transcription factor NF-κB inhibitor’ was positive.

### Patient recruitment and ethics

Samples used for the generation of human enteroids were donated by patients without evidence of IBD attending colonoscopy at the Royal Liverpool and Broadgreen University Hospitals NHS Trust (Liverpool, UK). Samples were collected following written informed consent and favourable ethical opinion from North West-Liverpool East Research Ethics Committee (15/NW/0045). All clinical investigation was conducted according to the principles expressed in the Declaration of Helsinki.

### Animal maintenance and welfare

All animal breeding, maintenance and procedures were performed under a UK Home Office licence, with local Animal Welfare and Ethics Review Board approval. Transgenic animals were maintained at the University of Manchester (Manchester, UK). Wild-type mice were purchased from Charles River (Margate, UK) and maintained either at the University of Manchester or University of Liverpool (Liverpool, UK) in specific pathogen-free facilities with access to standard chow and drinking water *ad libitum* unless otherwise specified. Standard 12-h light/dark cycles were used; standard temperature and humidity levels were maintained.

### Transgenic mouse strains

#### Human TNF luciferase mice (hTNF.LucBAC)

This line has been engineered to have a bacterial artificial chromosome (BAC)-expressing luciferase under the regulation of the entire human *TNF* promoter. Primary cultures established from this mouse enable direct measurement of *TNF* promoter activity in a non-transformed *ex vivo* system (Minshawi et al., 2019)*.*

#### Human p65-DsRedxp/IκBα-eGFP mouse (p65-DsRedxp/IκBα-eGFP)

This line expresses fusion proteins of NF-κB (p65) and the *Discosoma* red fluorescent protein – Express (DsRedxp) under the regulation of the native human p65 promoter, and IκBα and enhanced green fluorescent protein (eGFP), regulated by the human IκBα promoter (Dudek et al., 2017). Primary cultures established from this mouse enable direct visualisation of these fusion proteins*.*

### Peritoneal macrophage isolation, *in vitro* culture and luciferase assay

Resident peritoneal macrophages were obtained from mice by standard methods. Cells were stimulated with either 10 ng/ml LPS (*Salmonella enterica* serovar Minnesota R595; Calbiochem), 40 ng/ml TNF (R&D Systems), 10 µg/ml MDP (Invivogen) or 500 ng/ml flagellin (Novus Bio). Luciferase activity was measured over time in a CO_2_ luminometer (Lumistar Omega, BMG Labtech).

### Flow cytometry

Peritoneal macrophages were stimulated with 1 µg/ml LPS for 20 min. Fixed cells were stained for phosphor-p65 [pre-diluted PE Mouse anti-NF-κB p65 (pS529), BD Biosciences, clone K10-895.12.50] and analysed by flow cytometry using an LSRII Cytometer (BD Biosciences). Data analyses were performed with FlowJo887 software (Tree Star).

### Primary epithelial cell culture

Enteroids were generated from human and murine tissue using modifications of established protocols. Tissue samples were disaggregated by calcium chelation in EDTA followed by mechanical disaggregation in a sucrose/sorbitol solution. Crypt pellets were resuspended in Matrigel (Corning, UK) and were maintained in medium containing a combination of recombinant growth factors and growth factor-containing conditioned medium. Enteroids were passaged at least once prior to experiments.

### Wnt-3a conditioned medium

L Wnt-3a cells (ATCC^®^ CRL-2647™) were seeded into a T75 flask and passaged when ∼80% confluent into another T75 flask with 10 ml selection medium [Dulbecco's modified eagle's medium (DMEM) supplemented with 10% foetal calf serum (FCS), 2 mM penicillin-streptomycin and 300 µg/ml zeocin (Invivogen)]. When confluent, cells were passaged at a 1:4 split ratio into T150 flasks and 15 ml selection medium was added. After 24 h, cells were washed with PBS and the medium was changed to 10 ml reduced serum medium of DMEM supplemented with 5% FCS and 2 mM penicillin-streptomycin and grown until over-confluent (∼4-5 days). The medium was collected and centrifuged at 2000 ***g*** for 5 min at 4°C. The supernatant was filtered through a 0.22 µm syringe filter (Millipore) and stored at −80°C until use.

### Basal murine organoid medium

This medium consisted of Advanced DMEM/F12 medium (Sigma-Aldrich, Gillingham, UK) supplemented with 2 mM Glutamax-I (Thermo Fisher Scientific), 10 mM Hepes (Sigma-Aldrich), 1× N2 and B27 growth factors (both Invitrogen) and 1× antibiotic/antimycotic mixture (Thermo Fisher Scientific).

### Human seeding medium

The seeding medium contained 50% human basal medium and 50% Wnt-3a conditioned medium, with the addition of 50 ng/ml hEGF, 100 ng/ml hNOG, 1 µg/ml hR-spondin-1 (all R&D Systems), 1 mM N-acetylcysteine (Sigma-Aldrich), 10 mM nicotinamide (Sigma-Aldrich), 10 nM Gastrin (Bachem), 10 µM SB202190, 0.01 µM PGE2 and 0.5 µM LY2157299.

### Human organoid culturing medium

The organoid culturing medium contained 50% human basal medium and 50% Wnt-3a conditioned medium, with the addition of 50 ng/ml hEGF, 100 ng/ml hNOG, 0.5 µg/ml hR-spondin-1 (all R&D Systems), 10 mM nicotinamide (Sigma-Aldrich), 10 nM Gastrin (Bachem), 10 µM SB202190 and 0.01 µM PGE2.

### Human organoid culture

Five ileal biopsies were taken from non-IBD patients that were enrolled onto the SysMedIBD study and placed into 1 ml PBS containing 1× antibiotic-antimycotic (PBSAA) for transport to the laboratory. In a fume hood, biopsies were washed ten times in 2 ml PBSAA. Biopsies were placed in 4 ml ice-cold chelation buffer (3 mM EDTA in PBS) and incubated at room temperature for 30 min without agitation. Chelation buffer was discarded and replaced with 4 ml shaking buffer (43.3 mM sucrose and 59.4 mM sorbitol in PBS). Biopsies were mechanically agitated in the shaking buffer for 3 min or until the crypts were no longer seen on the biopsy surface. The crypt suspension was pelleted by centrifugation at 200 ***g*** for 5 min at 4°C, resuspended in 500 µl Matrigel (Corning, UK), and 50 µl was plated per well of a 24-well plate. Matrigel was polymerised at 37°C before applying 500 µl of human seeding medium per well. After 3 days, human seeding medium was changed to fresh human culturing medium, which was replaced every 3 days. Organoids were passaged every 7 days.

Terminal ileal organoids were passaged and cultured for 5 days before being removed from the Matrigel using 400 µl cell recovery solution (Corning) on ice for 40 min. The organoid suspension was transferred to a 15 ml Falcon tube and centrifuged at 200 ***g*** for 5 min. The organoids were washed in PBS and resuspended in fresh human culturing medium. Pre-treatment with or without 10 µM CLA or 1% DMSO (vehicle control) was applied in suspension for 30 min. Following pre-treatment, 100 ng/ml recombinant human TNF (Peprotech) was applied for a further 30 min and then fixed with 4% paraformaldehyde for 20 min. Fixed organoids were washed with PBS twice and transferred into 300 µl Richard-Allan™ Histogel™ (Thermo Fisher Scientific) and left to polymerise on ice for 30 min. Once set, the organoid-containing Histogel sample was processed as normal and paraffin embedded.

### Enteroid immunohistochemistry

Enteroids were pre-treated with 10 µM CLA or 1% v/v DMSO (vehicle) for 30 min. Following pre-treatment, 100 ng/ml recombinant human TNF (Peprotech) was applied for a further 30 min. Fixed enteroids were transferred into Richard-Allan™ Histogel™ (Thermo Fisher Scientific) and processed for histology. Immunohistochemical analysis was performed to visualise p65 (Cell Signaling Technology, #8242; 1:100).

Enteroids were selected for scoring based on size criteria; enteroids with fewer than 20 cells per circumference were excluded from analysis, and the average number of cells scored per enteroids was 118 (range 46-239). The proportion of cells nuclear stained for p65 was quantified for each organoid using manual cell counting by a scorer experienced in quantitative histological techniques (K.L.) and blinded to experimental conditions for each sample. First, for one circumference of an organoid, all nuclei were counted using a handheld tally counter. Immediately afterwards, the same section was scored for number of positively stained nuclei and the percentage of positively stained nuclei per organoid circumference calculated. For each patient, at least four enteroids were assessed, and there were six patients represented per group. Statistical testing was based on *N*=6 patients.

### Murine intestinal organoid confocal microscopy

Proximal enteroids from p65-DsRedxp/ IκBα-eGFP dual-reporter mice were passaged into glass-bottom dishes (Thermo Fisher Scientific™ Nunc™ Glass Bottom Dishes) in Phenol Red-free complete medium. Images were taken for six to eight enteroids per dish using a Zeiss Laser Scanning Microscope (LSM880) with a C-Apochromat 40×/1.2 W Korr FCS M27 objective. Enteroids were imaged for 30 min before treatment with 10 µM CLA or 1% v/v DMSO vehicle. Thirty minutes later, cultures were stimulated with 100 ng/ml TNF and imaged for a further 3 h. Images and videos were processed using Zen 2011 software and CellTracker (Warwick Systems Biology Centre).

### LPS-induced NF-κB activation in mice

Groups of adult (8- to 10-week-old) male C57BL/6 mice received 50 mg/kg CLA intraperitoneally (i.p.) or vehicle daily for 3 days. Following a 3-day washout period, either 50 mg/kg CLA i.p. or 0.9% w/v saline vehicle was administered; the next day either 50 mg/kg CLA i.p. or vehicle was administered followed by either 0.125 mg/kg ultrapure LPS from *E. coli* K12 (Invivogen) i.p. or 0.9% w/v saline vehicle. Animals were culled 90 min after LPS/vehicle administration; small intestinal mucosal scrapes were prepared in RIPA buffer with protease inhibitors (Sigma-Aldrich). NF-κB p65 DNA binding was quantified using a TransAM DNA-binding ELISA (ActiveMotif, La Hulpe, Belgium).

### DSS-induced colitis

Groups of adult (8- to 10-week-old) male C57BL/6 mice were administered either 10 mg/kg CLA or vehicle by orogastric gavage daily for 4 days. Following a washout period, animals received 2.5% DSS in drinking water, for 5 days. Animals recovered for a further 3 days. From the start of DSS treatment to termination of the experiment, either 10 mg/kg CLA or normal saline vehicle was administered by daily orogastric gavage. Tissue samples were harvested and prepared for histological analysis, including quantitative histology, as previously described (Williams et al., 2016).

### Statistical analysis

Statistical analyses were performed using GraphPad Prism v.7.0 software. Specific statistical tests are annotated in the text and figure legends. Reported *P-*values are two-tailed. Normality testing was performed with the D'Agostino and Pearson test.

## Supplementary Material

Supplementary information
