# Supplementary material for: Using systems medicine to identify a therapeutic agent with potential for repurposing in inflammatory bowel disease
Source: Dis Model Mech. 2020 Nov 27;13(11):dmm044040. doi: 10.1242/dmm.044040 (PMC7710021; doi:10.1242/dmm.044040)
Supplement: Supplementary information [file dmm-13-044040-s1.pdf]

Supplementary figures

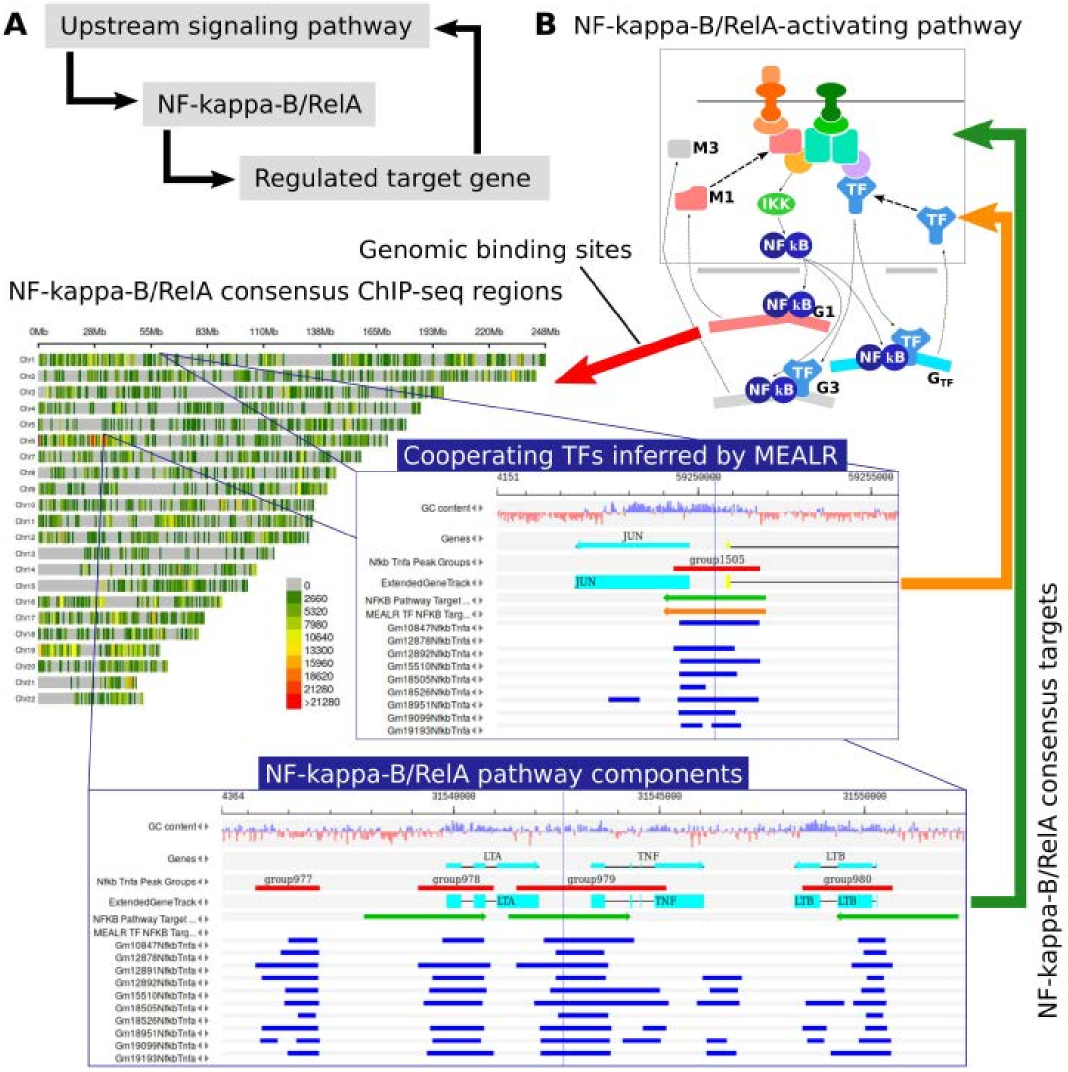

Disease Models & Mechanisms • Supplementary information

Fig. S1. (A) Schematic of the transcriptional feedback loops sought by our analysis. Upon activation through upstream signalling cascades the transcriptional regulator NF-kappa-B/RelA (and cooperating transcription factors) control expression of target genes which may themselves play a role in signalling cascades regulating the activity of NF-kappa-B/RelA thereby establishing a positive or negative feedback loop depending on whether NF-kappa-B/RelA enhances or interferes with the transcription of respective target genes. (**B**) More detailed illustration of identified feedback loops. Using consensus NF-kappa-B/RelA binding sites collected from 10 ChIP-seq experiments we analysed their genomic locations with respect to nearby potential target genes which were known components of relevant pathways and/or transcription factors whose motifs played a role for target sequence recognition as inferred by MEALR. Details of the compiled and analysed data are exemplified for pathway target genes TNF, LTA and LTB on chromosome 6 and for the cooperating transcription factor JUN on chromosome 1. The consensus peak density presentation was calculated using the CMplot package. The detailed views of genomic regions were created using the genome browser of the geneXplain platform.

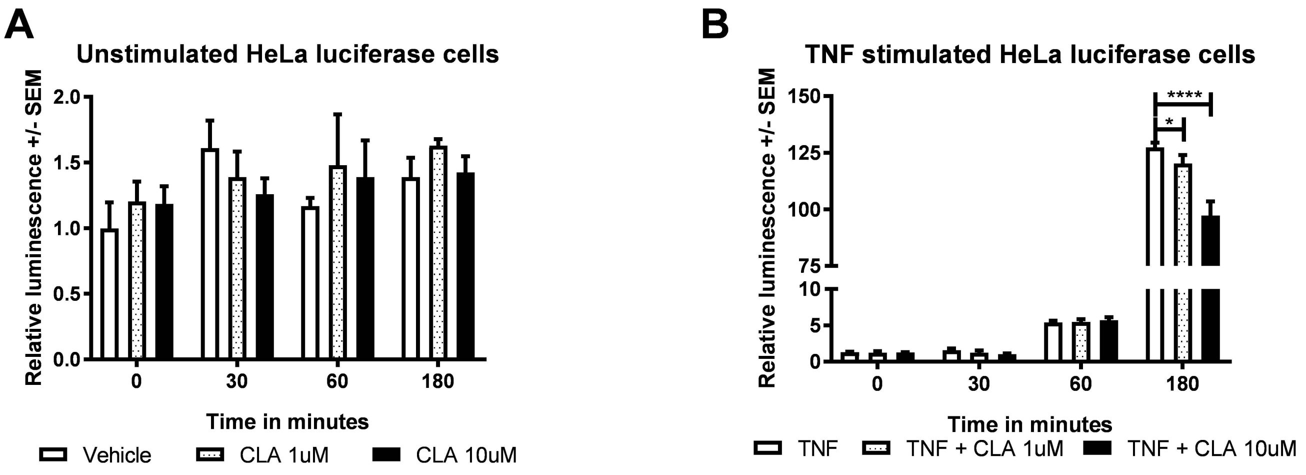

Fig. S2. Relative luciferase activity in unstimulated HeLa cells treated with DMSO vehicle (open bar), 1uM (hatched bar) or 10uM (solid bar) clarithromycin. B: Relative luciferase activity in TNF stimulated HeLa cells treated with DMSO vehicle, 1uM or 10uM CLA. * denotes p<.05, ****p<.0001, by 2-way ANOVA and Dunnett's posthoc test.

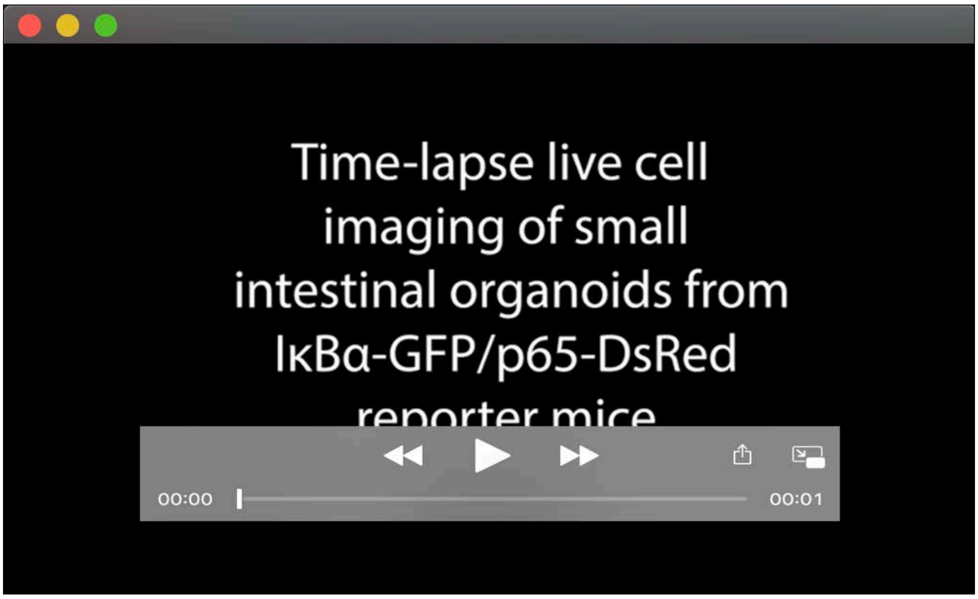

Movie 1. Representative timelapse confocal video of red channel images of dynamic, live-cell imaging studies of enteroids derived from p65-DsRedxp/ IκBalpha-eGFP mice, either treated with 100ng/mL TNF alone, or pre-treated with 10µM clarithromycin and subsequently stimulated with TNF.

Disease Models & Mechanisms • Supplementary information

Table S1. NF-κB co-operating transcription factors identified by MEALR

Click here to Download Table S1

Table S2. Interactions between genes and or proteins identified by text mining algorithm

Click here to Download Table S2

Table S3. Controlling node genes that may regulate signalling activity on the NF-κB network

Click here to Download Table S3

Table S4. IBD target genes, with source of data

Click here to Download Table S4

Table S5. List of IBD key-nodes, potential therapeutic targets for IBD

Click here to Download Table S5

Table S6. IBD key-nodes and their associated PASS activities

Click here to Download Table S6
